# Frequency Dependent Alterations in Regional Homogeneity of Baseline Brain Activity in Schizophrenia

**DOI:** 10.1371/journal.pone.0057516

**Published:** 2013-03-06

**Authors:** Rongjun Yu, Ming H. Hsieh, Hsiao-Lan Sharon Wang, Chih-Min Liu, Chen-Chung Liu, Tzung-Jeng Hwang, Yi-Ling Chien, Hai-Gwo Hwu, Wen-Yih Isaac Tseng

**Affiliations:** 1 Division of the Humanities and Social Sciences, California Institute of Technology, Pasadena, California, United States of America; 2 Department of Psychiatry, National Taiwan University Hospital and College of Medicine, Taipei, Taiwan; 3 Neurobiology and Cognitive Science Center, National Taiwan University, Taipei, Taiwan; 4 Center for Optoelectronic Biomedicine, National Taiwan University College of Medicine, Taipei, Taiwan; 5 Graduate Institute of Brain and Mind Sciences, National Taiwan University College of Medicine, Taipei, Taiwan; 6 Department of Medical Imaging, National Taiwan University Hospital, Taipei, Taiwan; University of Adelaide, Australia

## Abstract

Low frequency oscillations are essential in cognitive function impairment in schizophrenia. While functional connectivity can reveal the synchronization between distant brain regions, the regional abnormalities in task-independent baseline brain activity are less clear, especially in specific frequency bands. Here, we used a regional homogeneity (ReHo) method combined with resting-state functional magnetic resonance imaging to investigate low frequency spontaneous neural activity in the three different frequency bands (slow-5∶0.01–0.027 Hz; slow-4∶0.027–0.08 Hz; and typical band: 0.01–0.08 Hz) in 69 patients with schizophrenia and 62 healthy controls. Compared with controls, schizophrenia patients exhibited decreased ReHo in the precentral gyrus, middle occipital gyrus, and posterior insula, whereas increased ReHo in the medial prefrontal cortex and anterior insula. Significant differences in ReHo between the two bands were found in fusiform gyrus and superior frontal gyrus (slow-4> slow-5), and in basal ganglia, parahippocampus, and dorsal middle prefrontal gyrus (slow-5> slow-4). Importantly, we identified significant interaction between frequency bands and groups in the inferior occipital gyrus and caudate body. This study demonstrates that ReHo changes in schizophrenia are widespread and frequency dependent.

## Introduction

Schizophrenia is a devastating neuropsychiatric disorder which affects approximately 1% of the whole population [1], and is still one of the most mysterious and costliest mental disorders [2], [3]. Neuroscience tools have provided us more insight into the brain and have been a focus of schizophrenia research in recent years [4], showing that schizophrenia is a disorder of abnormal functional connectivity[5]–[11]. Recent studies have identified abnormal functional connectivity (FC) in the paradigm-free resting state in schizophrenia, including the default mode network [5], [12], the sensorimotor cortex, prefrontal cortex [13], [14], temporo-parietal area [15], thalamus [16], hippocampus [17], and amygdala [18].

Brain oscillations are important for understanding the cognitive impairment in schizophrenia [19]–[21]. Low frequency oscillations and their cross-frequency coupling are involved in cognitive function impairment in schizophrenia [20]. While functional connectivity can reveal the synchronization between distant brain regions, the local features of spontaneous brain activity in schizophrenia are still unknown. It is possible that schizophrenia may be associated with aberrant connectivity or synchronization within “local” regions as well as disturbed interactions between distinct regions.

Regional homogeneity (ReHo), which measures the similarity of the time series of a given voxel with its nearest neighbors within a single region, provides important information about the local temporal synchrony in the brain [22]. Since the BOLD (blood oxygenation level dependent) signal of functional MRI (fMRI) reflects neural activity [23], abnormal ReHo is probably related to changes in the temporal aspects of the spontaneous neural activity in the regional brain. It can be speculated that an abnormal ReHo may be a sign of disrupted local functionality. In fact, analysis of ReHo has been successfully used to detect local abnormality in subjects with different psychiatric disorders, including ADHD [24], [25], depression [26], [27], Parkinson’s disease [28], Alzheimer’s dementia [29], and schizophrenia [30]. A preliminary ReHo study with a small schizophrenia sample size (n = 18) reported that patients with schizophrenia exhibited decreased ReHo in widespread regions over the frontal, temporal, and right parietal cortex [30]. No increased ReHo was identified in that study. To date, most resting-state fMRI studies have examined spontaneous low frequency oscillation (LFO) activities in a specific frequency band of 0.01–0.1 Hz because the frequency band was thought to be linked to neuronal fluctuations [31]–[33]. Nonetheless, some researchers have observed that neuronal oscillations are distributed linearly on the natural logarithmic scale and that independent frequency bands are generated by distinct oscillators with specific properties and physiological functions [34], [35]. By decomposing LFOs of the BOLD signal into four distinct frequency bands [slow-5 (0.01–0.027 Hz), slow-4 (0.027–0.073 Hz), slow-3 (0.073–0.198 Hz), and slow-2 (0.198–0.25 Hz)], Zuo et al. (2010) showed that LFO amplitudes in the slow-4 band were higher than those in the slow-5 band in many brain regions, such as the basal ganglia, thalamus, and precuneus [36]. To our understanding, very few studies have investigated the effect of spatial frequency on ReHo. Here we sought to examine the ReHo in a relatively large patient sample (n = 69) and correlated the ReHo values with clinical measures.

We predicted that patients with schizophrenia would not only show decreased ReHo in regions associated with cognitive control, language, and memory, but also show increased ReHo in regions implicated in sensory-motor functions. Importantly, we examined ReHo in the slow-4 and slow-5 bands in addition to the frequency band (0.01–0.08 Hz) traditionally used to identify potential frequency-dependent changes.

## Materials and Methods

### Participants

From July 2009 to December 2010, 70 patients with DSM-IV diagnostic criteria of schizophrenia (34 males, 36 females) were recruited from outpatient clinics of National Taiwan University Hospital. Exclusion criteria included the presence of DSM-IV Axis I diagnoses of other disorders such as bipolar disorder, a history of any substance dependence, or a history of clinically significant head trauma. The positive and negative syndrome scale (PANSS) for schizophrenia was measured within one week before or after the MRI scan [37]. In the same period, 62 age-compatible healthy subjects were recruited as control group subjects. All of the control group subjects were free of the DSM-IV diagnoses of schizophrenia and other DSM-IV Axis I diagnoses of other disorders. None of them had neurological diseases, a history of any substance dependence, or a history of clinically significant head trauma. Written informed consent was obtained from participants, and our research procedures were followed by the Institutional Review Board of National Taiwan University Hospital (Approval series: 200809078R). The informed consent procedures included the standard consent form and all potential participants who declined to participate or otherwise did not participate were eligible for treatment (if applicable) and were not disadvantaged in any other way by not participating in the study. All patients were being treated with a range of antipsychotics (see Table S1 for details) with the most common being Abilify (aripiprazole, n = 20) or Zyprexa/ZyprexaZydis (n = 12).

Table 1 shows the sample demographics. For the schizophrenia group, one subject was removed because of his significant head movement during the experiment (movement greater than 3 mm of translation or 3° of rotation). As shown in the Table 1, the average age of our 69 schizophrenia patients was 31.7±9.6 years, and mean education was 14.2±2.1 years. In contrast, the average age of control subjects was 29.9±8.6 years, and their mean education was 15.3±2.4 years. All the data distributions were checked and there was no outlier in the measures. For the education parameter, the Wilcoxon-Mann-Whitney test suggested that there was a significant group difference in their personal educational attainment, *p* = .004. This was expected, as patients’ psychiatric disorders often hold back learning achievement. According to the Edinburgh Handedness Inventory [38], only one in the schizophrenia group and one in the control group were left-handed. The other subjects in both groups were right-handed. Demographic data also suggest our sample groups did not differ in gender distribution (*χ^2^* = 1.423, *p* = 0.233) or average age (*t* test, *p* = 0.264), as well as head motion during scanning (see Table 1 and Table S2).

**Table 1:** Sample Demographics.

Measure	Schizophrenia (n = 69)	Control (n = 62)	Statistics
	Mean (SD)	Mean (SD)	P
Age (year)	31.7 (9.6)	29.9 (8.6)	0.264
Education (year)^a^	14.2 (2.1)	15.3 (2.4)	0.004^b^
Illness duration (year)	7.1 (6.5)	n.a.	n.a.
PANSS-positive scale (n = 64)	12.1 (4.7)	n.a.	n.a.
PANSS-negative scale (n = 64)	13.4 (6.1)	n.a.	n.a.
GANSS-general scale (n = 64)	27.4 (9.6)	n.a.	n.a.
PANSS-total (n = 64)	52.9 (16.8)	n.a.	n.as.

Note: Demographic information for the patient sample and control sample. Mean and standard deviation are provided for continuous variables (e.g., age, education, and PANSS scales). PANSS = Positive and Negative Syndrome Scale.

a *p*<.005;

b the Wilcoxon Mann Whitney test (two tailed *p*-value).

### MRI Data Acquisition

All subjects underwent structural and functional MRI scans in a single session using a 3T MR system (TIM Trio, Siemens, Erlangen, Germany). A 32-channel head coil was used as the RF signal receiver. Sponges were used to fix the subject’s head within the coil to prevent motion artifacts. All images were acquired parallel to anterior-commissure-posterior-commissure line with an auto-align technique. The total scan time for each subject was about 10 minutes.

T2-weighted images were acquired with a turbo spin-echo sequence. The image parameters were as follows: TR/TE = 7240 ms/101 ms, FOV = 240 mm × 240 mm, matrix = 256 × 256, slice thickness = 3.5 mm, and flip angle = 150°. The GRAPPA technique with an acceleration factor of two was used. A total of forty three contiguous axial slices were acquired in approximately 2 minutes. For the reference image of anatomy, a magnetization-prepared rapid gradient echo (MPRAGE) sequence was used to acquire a whole brain high-resolution T1-weighted MR image in a coronal view. The sequence parameters were TR/TE = 2000 ms/2.98 ms, inversion time = 900 ms, image matrix size = 192×256, spatial resolution = 1 mm×1 mm, field of view (FOV) = 192 mm × 256 mm, and slice thickness = 1 mm without gaps. T1 scan time was about 3 min and 36 sec.

The resting state fMRI was performed with a gradient-echo echo planar imaging sequence. Subjects were asked to relax and think of nothing in particular with eyes closed but were requested not to fall asleep. Wakefulness was assessed throughout the recording via an intercom link to the scanner chamber. The fMRI acquisition parameters were as follows: TR/TE = 2000 ms/24 ms, FOV = 256 mm × 256 mm, matrix = 64 × 64, slice thickness = 3 mm, interleaved scanning, and flip angle = 90°. For each participant, thirty-four trans-axial slices with no gap were acquired to encompass the whole brain volume. The scan time of the resting-state fMRI was approximately 6 minutes.

### Image Processing

The first 10 volumes of each functional time series were discarded because of instability of the initial MRI signal and adaptation of participants to the circumstance, leaving 170 volumes in total. The remaining fMRI images were slice acquisition corrected, head-motion corrected (a least squares approach and a 6-parameter spatial transformation), normalized to the standard SPM5 Montreal Neurological Institute (MNI) template, and then re-sampled to 3-mm cubic voxels. Subjects with head motion more than 3.0 mm of maximal translation (in any direction of x, y or z) or 1.0° of maximal rotation throughout the course of scanning were excluded from further analysis. After linear detrending, data was filtered using typical temporal bandpass (0.01–0.08 Hz), slow-5 bandpass (0.01–0.027 Hz), and slow-4 bandpass (0.027–0.073 Hz) separately. Six motion parameters, the cerebrospinal fluid (CSF), the global mean signal, and the white matter signals as nuisance covariates to reduce the effects of head motion and non-neuronal BOLD fluctuations [39], [40]. Regional homogeneity analysis was performed for each participant by calculating the Kendall’s coefficient of concordance (KCC) of the time series of a given voxel with those of its nearest neighbours (26 voxels) in a voxel-wise analysis:
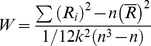



where W ranges from 0 to 1; 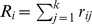 where r*_ij_* is the rank of the *i* ^th^ time point in the *j* ^th^ voxel; 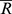 = (n+1)k/2 is the mean of the R*_i_*, n is the number of time points of each voxel time series (here n = 170), and k is the number of time series within the measured cluster (here k = 27, the central voxel plus its 26 neighbours). The intracranial voxels were extracted to make a mask. For standardization purposes, each individual ReHo map was divided by its own mean ReHo within the mask. The resulting fMRI data were then spatially smoothed with a Gaussian kernel of 6×6×6 mm^3^ full-width at half-maximum.

To explore the ReHo differences between the groups, a second-level random-effect two-sample t-test was performed on the individual normalized ReHo maps in a voxel-by-voxel manner. A threshold of family-wise error (FWE) corrected threshold of p<0.05 at the cluster level was set. All coordinates are reported in MNI coordinates, as used by SPM. The regions that showed significant differences were then extracted as regions of interest (ROI) and the beta values in peak voxels were used for a post hoc analysis. In order to determine whether the ReHo value in each region varied with clinical measures, correlation analyses between the beta values from peak voxels and each of the clinical variables (e.g., PANSS scores and illness duration) were performed using a 2-tailed α level of 0.05.

## Results

### ReHo in Typical Frequency Band (0.01–0.08 Hz)

**Figure 1:**
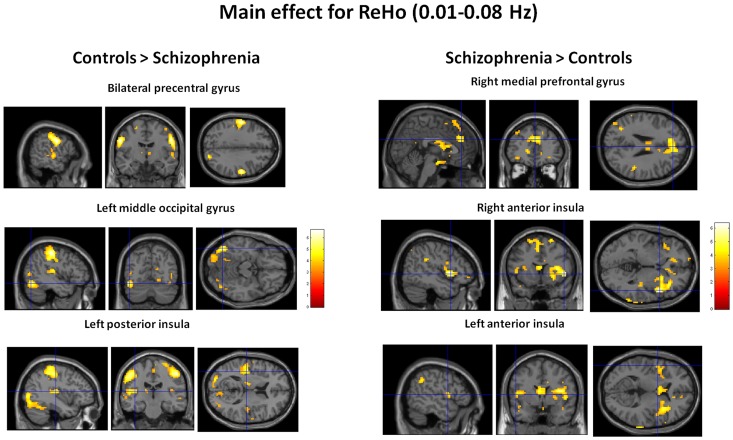
Regions that showed a significant ReHo difference between patients with schizohprenia and controls: lower ReHo in the bilateral precentral gyrus, left middle occipital gyrus, and left posterior insula; higher ReHo in the right medial prefrontal gyrus and bilateral anterior insula. For display purposes only, all statistical maps (P<0.001, uncorrected) are overlayed on a T1-weighted MNI template.

We first reported ReHo results from the typical frequency band (0.01–0.08 Hz). Group differences are shown in Table 2 and Figure 1. Compared to controls, patients exhibited reduced ReHo in the bilateral precentral gyrus, left middle occipital gyrus, and left posterior insula. Patients had higher ReHo in the right medial prefrontal gyrus and bilateral anterior insula. No significant correlations between ReHo and clinical measures reached significance (P values >0.1).

**Table 2:** Brain regions showing differences in the regional homogenity (ReHo, 0.01–0.08 Hz) between controls and patients with schizohrenia.

Bain regions	Z scores	Voxels	X	Y	Z
Control *vs.* Schizophrenia					
**L Precentral gyrus**	6.17	1926	−57	−9	33
R Precentral gyrus	6.17		51	−21	51
**L Middle occipital gyrus**	5.95	977	−45	−69	−15
R Precuneus	4.96		21	−84	36
**L Posterior insula**	5.24	173	−39	−21	12
L Transverse temporal gyrus	4.67		−51	−21	9
Schizophrenia *vs.* Control					
**R Medial prefrontal cortex**	4.67	1071	3	42	27
L Medial prefrontal cortex	4.56		−9	36	18
**R Anterior insula**	5.93	604	48	12	0
**L Anterior insula**	4.22	138	−51	3	9

Note: X, Y and Z are MNI coordinates.

### Frequency-dependent Effects

**Figure 2:**
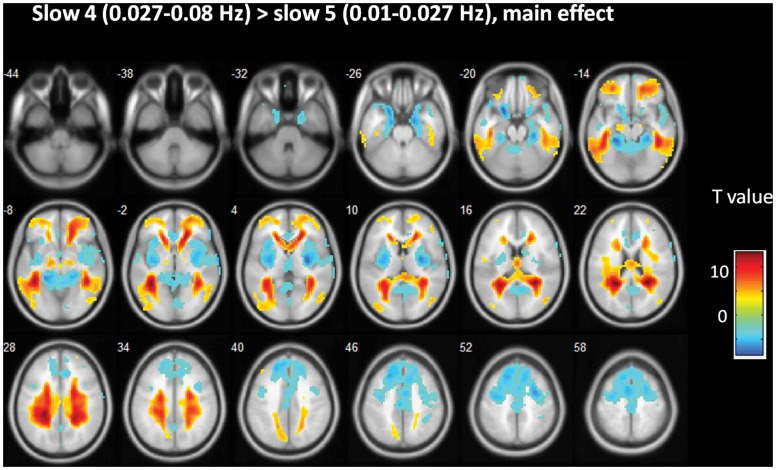
The main effect for frequency band on ReHo. Hot colors represent higher ReHo in the slow-4 band than in the slow-5 band, whereas blue cold colors represent lower ReHo. The results were obtained by a two-way repeated-measures ANOVA.

Next, we examined the effect of frequency band on ReHo. Main effects from the two-way repeated-measures ANOVA are shown in Fig 2. Brain regions showing a significant larger ReHo in slow 4 band than in slow 5 band were identified in the fusiform gyrus, and superior frontal gyrus, whereas larger ReHo in slow-5 were found in the culmen, parahippocampal gyrus, putamen, and dorsal middle prefrontal gyrus (Figure 2).

**Figure 3:**
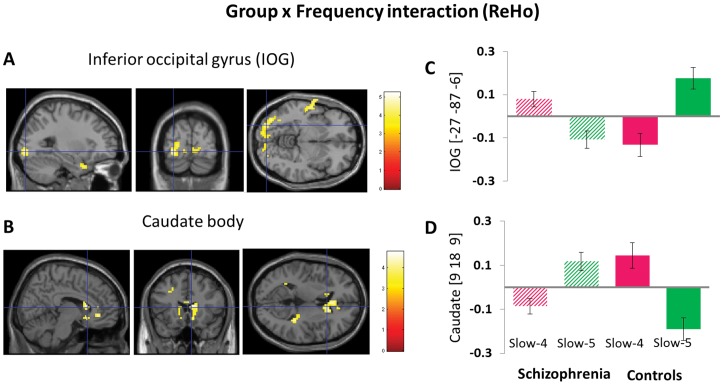
The interaction between frequency band and group on ReHo. The results were obtained by a two-way repeated-measures ANOVA and post-hoc test (A-B). The beta values for the left inferior occipital gyrus (IOG) and right caudate body were plotted (C-D) to show the directions of the interaction.

The main effect of group identified brain regions was consistent with those reported in previous analysis using the typical frequency band (0.01–0.08 Hz); see Table 3 for details. We also observed significant interaction between frequency band and group in the left inferior occipital gyrus and right caudate body (Figure 3 and Table 4). Beta values were plotted to illustrate the directions of these interactions (Figure 3).

**Table 3:** Brain regions showing differences in the regional homogenity (ReHo) between controls and patients with schizohrenia from the main effect of group.

Bain regions	Z scores	Voxels	X	Y	Z
Control *vs.* Schizophrenia					
**L Precentral gyrus**	6.24	1837	−57	−9	33
R Precentral gyrus	5.76		51	−21	51
**L Middle occipital gyrus**	5.94	1457	−45	−69	−15
R Precuneus	5.38		21	−84	36
**L Posterior insula**	4.46	185	−39	−21	12
L Transverse temporal gyrus	4.31		−51	−21	9
Schizophrenia *vs.* Control					
**R Medial prefrontal cortex**	4.29	224	3	42	27
L Medial prefrontal cortex	3.99		−9	36	18
**R Anterior insula**	5.05	326	48	12	0
**L Anterior insula**	4.22	138	−51	3	9

Note: X, Y and Z are MNI coordinates.

**Table 4:** Brain regions showing significant interaction in the ReHo between group and frequency band.

Bain regions	Z scores	Voxels	X	Y	Z
**L Inferior occipital gyrus**	5.06	346	−27	−87	−6
**R Caudate body**	4.54	177	9	18	9

Note: X, Y and Z are MNI coordinates.

## Discussion

Using the ReHo method, our study found that compared with controls, patients with schizophrenia exhibited significantly reduced ReHo in bilateral precentral gyrus, left middle occipital gyrus, and left posterior insula, as well as significantly increased ReHo in the right medial prefrontal cortex and bilateral anterior insula during the resting state. ReHo in the slow-4 band were higher in the fusiform gyrus, and superior frontal gyrus, and lower in the culmen, parahippocampal gyrus, putamen, and dorsal middle prefrontal gyrus, in comparison to ReHo in slow-5. We also found that ReHo in the inferior occipital gyrus and caudate body exhibited a significant interaction between frequency band and group. Our findings demonstrate that schizophrenia is associated with abnormal coherence of spontaneous neural activity in the regional brain, and these abnormalities are modulated by the frequency band.

Our study replicates the findings of Liu et al. [30] that patients with schizophrenia exhibited decreased ReHo in regions including the precentral gyrus and middle occipital cortex. Cortical volume reduction in the precentral gyrus has been found in schizophrenia [41]. The precentral gyrus may contribute to the processing of multiple motor-related cognitive functioning, which has been found to be abnormal in schizophrenia [42], [43]. Cortical thinning and gray matter density and volume reduction were also found in the occipital cortex of patients with schizophrenia [44]–[46]. The occipital cortex may be a neurobiological substrate of some of the deficits observed in early visual processing in schizophrenia, such as visual hallucinations and object-recognition deficits [45], [47].

However, we did not find any significant decrease in ReHo in regions identified in the previous study, such as the cerebellum, brainstem, and parahippocampal areas. One possible explanation is that the previous study recruited only 18 patients, which may have resulted in false positive findings. It is also worth noting that the characteristics of patients with schizophrenia, regarding age and illness duration, were different between the two ReHo studies. Schizophrenia patients in Liu et al.’s study were younger (mean age = 23.7±4.4 years) and had shorter illness duration (26.8±19.2 months) in comparison with those in our sample. Further investigations using ReHo methods are needed to clarify these inconsistent findings.

In contrast to the previous study, which reported no significant increase in ReHo in schizophrenia, we also found increased ReHo in the medial prefrontal cortex and anterior insula in schizophrenia. Increased ReHo in the medial prefrontal cortex seems to contradict the prior functional imaging finding of hypofrontality [48]–[55]. One possible reason is that the reliability of hemodynamic hypofrontality in schizophrenia seems controversial. A recent argument in task-related fMRI is whether task-related hypofrontality in schizophrenia represents an intrinsic cerebral malfunction – in other words, a subtle alteration of biological lesion – or whether it merely reﬂects the fact that schizophrenia patients typically have poorer cognitive performance and so activate their functional areas to a correspondingly lesser degree. Supporting this possibility, Frith et al. found that when patients with schizophrenia and healthy controls were matched for performance by the use of a paced form of verbal ﬂuency task, no significant results showing hypofrontality were found [56]. Also, a number of current studies have found evidence of increased activation in the prefrontal cortex in schizophrenia during performance of working memory tasks [57]–[60]. Schizophrenia patients in fact exhibit a failure of task-related deactivation in the medial frontal cortex [11], [61]. Our finding of enhanced ReHo in medial PFC in schizophrenia in the resting state may help explain the hyperfrontality in the task state.

The anterior insula integrates external sensory input with the limbic system and is integral to the awareness of the body’s state, such as identifying self-generated from externally-generated sensory information [62]–[64]. Insular volume reduction has consistently been found in schizophrenia [65]–[68]. Functional neuroimaging studies have shown that compared to healthy controls, patients with schizophrenia have an increased response in the left insula when attending to speech prosody [69]. Abnormal ReHo activity in the insula may contribute to their disruption in sensory-affect processing in schizophrenia and lead to internally generated sensory information being attributed to an external source, therefore contributing to hallucinations in schizophrenia.

There were significant interactions between frequency band and group in several regions, including the inferior occipital gyrus and caudate body. These findings indicate that ReHo changes in schizophrenia are frequency dependent, although the nature of these interactions is not clear and awaits further investigation. Using the typical frequency band may miss these new findings, and such frequency specific ReHo could carry important information for disease diagnosis and our understanding of the pathology of schizophrenia.

Some limitations in our study are worth mentioning. First, we did not collect physiological measures (respiration, heart rate), which may contribute to low-frequency BOLD fluctuations [70]–[73]. Moreover, it is possible that LFOs in a specific frequency band are particularly sensitive to physiological noises. Future studies may use simultaneous cardiac recording and regress out these facts to minimize the impact of the noises or develop better methods to reduce such noise (e.g. train participants to breathe regularly). Second, the origin and functional significance of ReHo are not yet clear, which limits the implication of ReHo group differences. Furthermore, it is still unknown whether and how ReHo abnormalities contribute to inter-regional functional connectivity and other large scale brain networks. Future studies are necessary to examine ReHo together with other LFO measures (e.g. amplitudes of LFO and functional connectivity) to acquire better understanding of their associations. It would also be interesting to study ReHo in both resting and task-based fMRI and examine whether ReHo, which represents baseline neural activity in local regions, predicts task induced signal changes in those regions. Finally, the nature of these data does not allow us to establish causal links. Prospective studies are needed to assess whether ReHo deficits are a cause or a consequence of schizophrenia.

In conclusion, this study not only revealed a typical decrease of ReHo in the precentral gyrus, middle occipital gyrus, and posterior insula, but also an atypical increase of ReHo in the medial prefrontal cortex and anterior insula. Our findings of increased ReHo in schizophrenia might suggest an altered coherence of local resting-state activity in brain regions responsible for high cognitive function. Furthermore, we first showed that ReHo varied with frequency bands, presenting slow-4> slow-5 in the fusiform gyrus and superior frontal gyrus, and slow-5> slow-4 in basal ganglia, parahippocampus, and dorsal middle prefrontal gyrus. We also demonstrated that ReHo abnormalities in schizophrenia were frequency-dependent in the inferior occipital gyrus and caudate body. Our work may carry potential implications for neural psychopathology of schizophrenia. Future work will be needed to examine the functional significance of these interactions (e.g., frequency band×group) and test similar frequency dependent changes in other psychiatric disorders.

## Supporting Information

Table S1 **Treatment details of schizophrenia patients.**
(DOC)

Table S2 **Participant motion parameters during fMRI scanning.**
(DOC)
