# Supplementary material for: Frequency Dependent Alterations in Regional Homogeneity of Baseline Brain Activity in Schizophrenia
Source: PLoS One. 2013 Mar 6;8(3):e57516. doi: 10.1371/journal.pone.0057516 (PMC3590274; doi:10.1371/journal.pone.0057516)
Supplement: Table S1 — Treatment details of schizophrenia patients. (DOC) [file pone.0057516.s001.doc]

**Table S1： Treatment details of schizophrenia patients**

| Treatment | Case number |
| --- | --- |
| Abilify | 20 |
| ZyprexaZydis | 8 |
| Risperdal | 5 |
| Prozac | 5 |
| Zyprexa | 4 |
| Invega | 4 |
| Solian | 4 |
| Flurazin | 4 |
| Seroquel | 4 |
| Dogmatyl | 4 |
| Seroxat | 3 |
| Zoloft  Other | 2  2 |
