# Supplementary material for: Frequency Dependent Alterations in Regional Homogeneity of Baseline Brain Activity in Schizophrenia
Source: PLoS One. 2013 Mar 6;8(3):e57516. doi: 10.1371/journal.pone.0057516 (PMC3590274; doi:10.1371/journal.pone.0057516)
Supplement: Table S2 — Participant motion parameters during fMRI scanning. (DOC) [file pone.0057516.s002.doc]

**Table S2. Participant motion parameters during fMRI scanning**

|  | *Schizo (n = 69)* | *HC (n = 62)* |  |
| --- | --- | --- | --- |
| Gender | 34 M / 35 F | 25 M / 27 F |  |
| Motion parameters | mean ± SD | mean ± SD | *P value* |
| X (mm) | 0.0036 ± 0.0239 | 0.0039 ±0.0383 | .967 |
| Y (mm) | 0.0086 ± 0.0936 | 0.0045 ±0.0905 | .804 |
| Z (mm) | -0.0728 ± 0.1766 | -0.0427 ±0.0799 | .226 |
| Pitch (。) | 0.0213 ± 0.0852 | 0.0214 ±0.0714 | .991 |
| Roll (。) | -0.0095 ± 0.0452 | -0.0025 ±0.0521 | .416 |
| Yaw (。) | 0.0039 ± 0.0407 | 0.0029 ±0.0324 | .868 |

Notes: SD - standard deviation; Motion parameters - six motion parameters (translation: x, y and z in mm; rotation: pitch, roll and yaw in degrees) were obtained from head movement correction for each participant.
